# ST Elevation in a Patient With COVID-19 Infection-Associated Fever: A Case of Brugada Pattern

**DOI:** 10.7759/cureus.8722

**Published:** 2020-06-20

**Authors:** Guruprasad Mahadevaiah, Abdul Aleem, Antonio Secaira, Samir Saba, Nasir Shariff

**Affiliations:** 1 Pediatric Cardiology, California Northstate University College of Medicine, Sacramento, USA; 2 Internal Medicine, Lehigh Valley Hospital, Allentown, USA; 3 Cardiology, Franciscan Heart and Vascular Associates, Tacoma, USA; 4 Cardiology, University of Pittsburgh Medical Center, Pittsburgh, USA

**Keywords:** brugada pattern, st-segment elevation myocardial infarction (stemi), coronavirus infection, covid-19, electrocardiogram

## Abstract

Coronavirus disease 2019 (COVID-19) is a global pandemic presenting with various cardiovascular manifestations. Although Brugada pattern ST-segment elevation (STE) is well described in patients admitted with febrile illness, the implication of recognizing this abnormality in patients with COVID-19 is critical in providing appropriate care for the patient and also reducing the exposure of healthcare professionals to the risk of infection. We report a patient with COVID-19 infection presenting with STE due to fever-related unmasking of Brugada pattern, who was managed conservatively.

## Introduction

Percutaneous coronary intervention improves outcomes in patients with acute ST-segment elevation (STE) myocardial infarction [1]. Brugada pattern of STE is often misinterpreted as STE myocardial infarction resulting in inappropriate invasive studies. Prompt identification of the Brugada pattern of STE in patients with coronavirus disease 2019 (COVID-19) infection-associated fever could reduce invasive procedures and also the exposure of healthcare professionals to the risk of infection. We report a patient with COVID-19 infection presenting with STE due to fever-related unmasking of the Brugada pattern.

## Case presentation

A 40-year-old male with no prior significant medical history presented to the hospital with fever and chest pain of two-day duration. He described the chest pain as left sided and worse with lying down. There was no significant family history of sudden cardiac death or premature coronary artery disease. He denied history of tobacco use. Two of his family members had been tested positive for COVID-19 infection a week prior. On physical examination, his temperature was 39.1°C (102.3°F), blood pressure (BP) 131/88 mmHg, heart rate (HR) 95 beats/min, respiratory rate (RR) 18 breaths/min, and SaO_2_ 95% on room air.

Initial laboratory and radiologic investigation, which included complete blood count, comprehensive metabolic panel, and chest X-ray, was within normal limits. Initial troponin level was normal. D-Dimer was 224 ng/mL (0-243 ng/mL).

**Figure 1:**
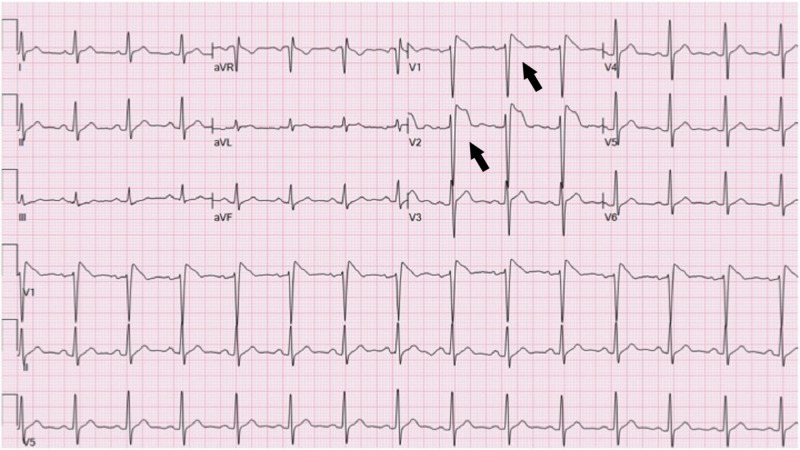
Electrocardiogram on initial presentation demonstrating Brugada type I pattern of ST-segment elevation in leads V1 and V2.

Electrocardiogram (EKG) demonstrated a right bundle branch block morphology with a coved pattern of STE of 5 mm in the right precordial chest leads (Figure 1). The patient was initiated on heparin infusion, and cardiology was consulted. Considering the clinical history and EKG suggestive of Brugada pattern, a decision was made to hold off on any invasive procedures. The patient received antipyretics and was further evaluated with an echocardiogram.

**Figure 2:**
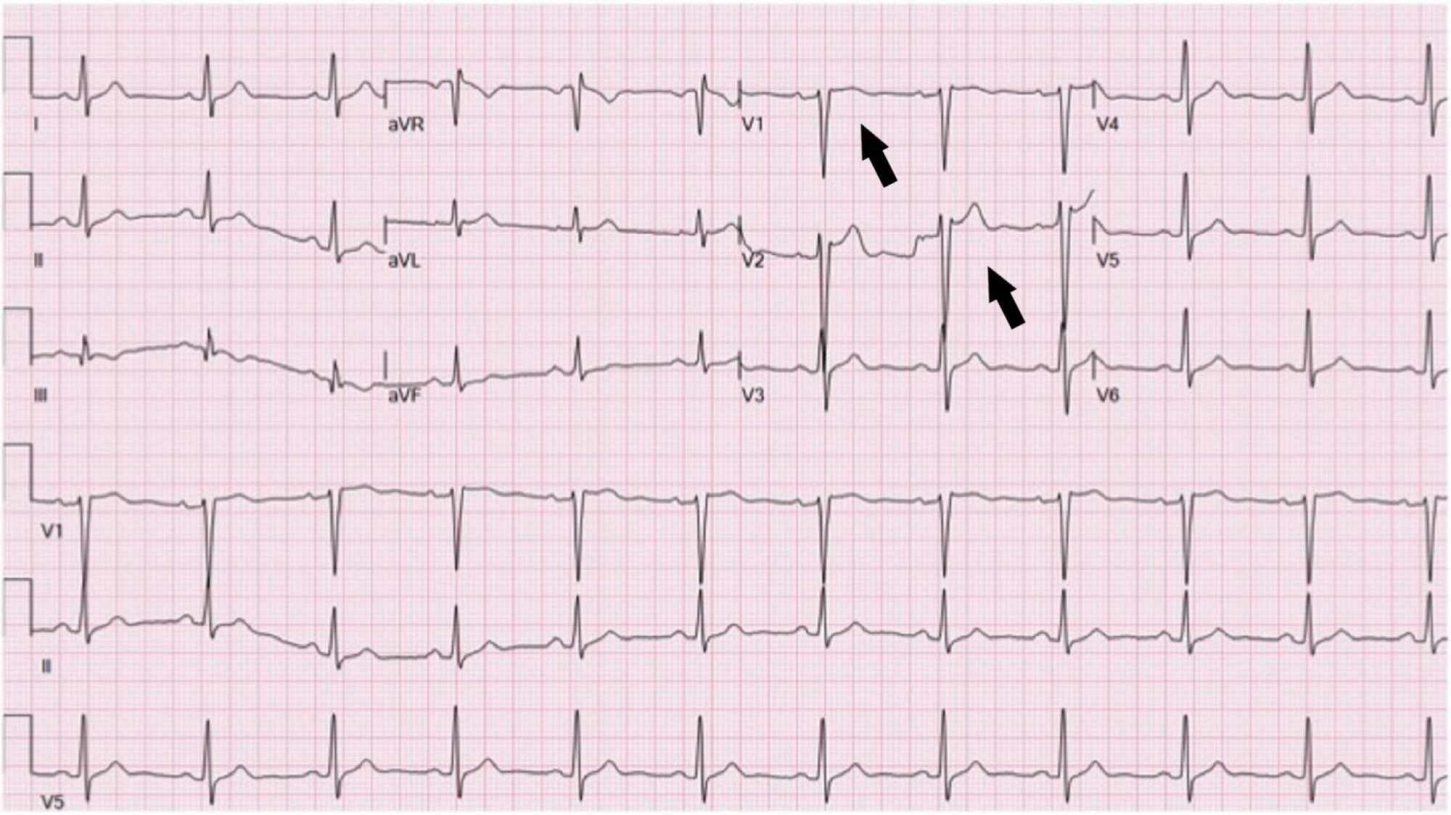
Electrocardiogram demonstrating significant resolution of ST-segment elevation following management of fever.

Echocardiogram demonstrated normal left ventricular (LV) function with no regional wall motion abnormality. Repeat EKG after 24 hours showed a significant resolution of the STE (Figure 2). Serial troponins were within normal limits. The patient was discharged the next day with information regarding isolation measures. 

## Discussion

Higher body temperatures result in reduced efficiency of the sodium channels presenting as Brugada pattern of STE in patients with a heterozygous mutation of sodium channels [2]. Patients with precipitated Brugada pattern without a history of syncope have a low risk of cardiac events [3]. Brugada pattern can be misinterpreted as STE myocardial infarction resulting in inappropriate invasive investigations. In patients presenting to the hospital with febrile illness, 2% were noted to have type I Brugada EKG pattern (coved pattern of STE of >2 mm with symmetric negative T wave in the right precordial chest leads) [4]. Fever is the predominant clinical feature of patients admitted with COVID-19 [5]. In a case series study of patients with COVID-19 who had STE indicating potential acute myocardial infarction, nine of the 18 patients had undergone coronary angiogram procedure of which four patients were reported to have nonobstructive disease [6]. There have been two case reports of the Brugada pattern of EKGs in patients infected with COVID-19 [7,8]. The first published case report was a 49-year-old male admitted after an episode of syncope and EKG showed Brugada pattern and a positive COVID-19 test [7]. The second published case report was a 61-year-old male who presented with subjective fever, shortness of breath, chest pain, and noted reduced global LV function [8]. Due to underlying high-risk factors, patients in both reports underwent coronary angiogram procedure that demonstrated normal coronary anatomy.

Although coronary intervention significantly reduces the ischemia time and improves outcomes in patients with acute STE myocardial infarction, patients presenting with STE during the COVID-19 pandemic pose a challenging situation. Given the droplet transmission of COVID-19, invasive interventions are associated with a high risk of exposure of healthcare professionals to the virus. Also, with the positive pressure ventilation settings used in catheterization labs, there is a theoretical risk of further widespread contamination. Prompt identification of Brugada pattern in patients with febrile illness due to COVID-19 infection could enable timely appropriate care, reduce the risk of unnecessary invasive procedures, and protect healthcare professionals from the risk of exposure to the virus. Our case illustrates that fever due to COVID-19 infection can unmask Brugada pattern ST elevation, and patients can be managed with supportive care and antipyretics for the noted cardiac findings.

## Conclusions

Our case illustrates that fever due to COVID-19 infection can unmask Brugada pattern ST elevation. Identification of the condition could result in timely appropriate care, reduce unnecessary invasive procedures, and protect healthcare professionals from the risk of infection.
